# Improvement of the Thermo-Oxidative Stability of Biobased Poly(butylene succinate) (PBS) Using Biogenic Wine By-Products as Sustainable Functional Fillers

**DOI:** 10.3390/polym15112533

**Published:** 2023-05-31

**Authors:** Benedikt T. Hiller, Julia L. Azzi, Mirko Rennert

**Affiliations:** 1 Institute for Biopolymers (ibp) at Hof University, Hof University of Applied Sciences, 95028 Hof, Germany; 2 Plastics Technology Group, Faculty of Mechanical Engineering, Technische Universität Ilmenau, 98683 Ilmenau, Germany; 3 Medical and Biological Physics Program, Faculty of Science, McMaster University, Hamilton, ON L8S 4LD, Canada; azzij@mcmaster.ca

**Keywords:** wine grape pomace, biogenic by-products, natural stabilizers, thermo-oxidative degradation, poly(butylene succinate), natural antioxidants, biocomposites

## Abstract

Biobased poly(butylene succinate) (PBS) represents one promising sustainable alternative to petroleum-based polymers. Its sensitivity to thermo-oxidative degradation is one reason for its limited application. In this research, two different varieties of wine grape pomaces (WPs) were investigated as fully biobased stabilizers. WPs were prepared via simultaneous drying and grinding to be used as bio-additives or functional fillers at higher filling rates. The by-products were characterized in terms of composition and relative moisture, in addition to particle size distribution analysis, TGA, and assays to determine the total phenolic content and the antioxidant activity. Biobased PBS was processed with a twin-screw compounder with WP contents up to 20 wt.-%. The thermal and mechanical properties of the compounds were investigated with DSC, TGA, and tensile tests using injection-molded specimens. The thermo-oxidative stability was determined using dynamic OIT and oxidative TGA measurements. While the characteristic thermal properties of the materials remained almost unchanged, the mechanical properties were altered within expected ranges. The analysis of the thermo-oxidative stability revealed WP as an efficient stabilizer for biobased PBS. This research shows that WP, as a low-cost and biobased stabilizer, improves the thermo-oxidative stability of biobased PBS while maintaining its key properties for processing and technical applications.

## 1. Introduction

**Figure 1:**
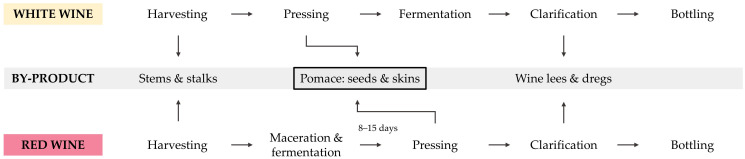
Main stages of the winemaking process with the corresponding by-products generated.

Bioeconomic approaches are garnering widespread attention in order to develop sustainable sources of raw materials by valorization of existing biomass. In particular, regional concepts are favorable to further reducing emissions by using local material streams. An agricultural industry that exists in various regions of the world is viticulture. Wine has been produced for over 6000 years in human history [1]. With the integration of industrial machinery to increase efficiency, the processing of grapes into wine has evolved, but the main process steps have remained the same. As illustrated in Figure 1, various by-products are generated during winemaking. In total, these by-products represent about one third of the initial grapes [2,3]. After harvesting, most of the stems and stalks are separated from the grapes [4]. While white grapes are subsequently pressed to obtain the grape juice for fermentation, red grapes are macerated and fermented for several days prior to pressing [5]. Through pressing, the (macerated) grape juice is separated from the wine grape pomace (WP). The by-product, WP, mainly consists of grape seeds and skins, with some remaining stems not fully separated during destemming [6]. It can be declared the main winemaking by-product, representing about 20–30 wt.-% of the initial grapes [5]. Accordingly, over 10 Mt of WP are produced worldwide every year [7]. Only a few percent of these large quantities of biomass are reused in the animal feed industry or to produce distilled spirits [6,8]. Despite containing valuable bioactive compounds, most of the by-product is declared agricultural waste, facing challenges in terms of limited disposal options and handling due to high moisture of up to 80% [9,10,11].

Multiple studies have been conducted to determine the composition of WP and its antioxidant activity and reevaluate its potential for valorization [5,12,13,14]. It is well-known that by-products of the winemaking industry—and especially WP—are rich in functional compounds; e.g., phytochemicals such as polyphenols [4,15]. Studies have revealed that about 70% of the grape phenolic compounds remain in WP [13,16]. Polyphenolic compounds are classified as flavonoids and non-flavonoids. The flavonoids present in WP are flavonols, such as rutin, kaempferol, and quercetin; flavan-3-ols, such as catechin and its chiral isomer epicatechin; and anthocyanins, such as malvidin and condensed tannins. Non-flavonoids in WP include stilbenes, such as resveratrol, as well as phenolic acids, such as chlorogenic acid and gallic acid [5,12,13,16,17,18]. Although WP generally shows high antioxidant potential, the exact chemical composition and antioxidant activity (e.g., radical scavenging activity) depend on the grape variety, the winemaking process, and the region and weather conditions of cultivation [8,19,20]. 

Due to their high potential for use as comparatively cheap and highly sustainable bio-additives and bio-fillers, research on the use of by-products from the winemaking industry in the field of plastics and polymer processing has been conducted in recent years [21]. In conventional petroleum-based polyolefins, such as polypropylene (PP) and polyethylene (PE), wine by-products and their extracts were able to improve the stability of the matrix polymers to suppress oxidative degradation and (UV-)aging [11,22,23]. As far as the combination of wine by-products and biopolymers is concerned, previous studies have focused either on the use of the by-products as low-cost, non-functional fillers or on the use of elaborately prepared extracts as stabilizers. Gowman et al. [24] incorporated WP as a filler in poly(lactic acid) (PLA) and developed a model to predict the mechanical properties of the resulting biocomposites. Monari et al. [4] performed an extraction procedure on WP to obtain functional compounds usable in different applications. With respect to the full valorization of the by-product, the solid extraction residues were used as fillers for poly(3-hydroxybutyrate-co-3-hydroxyvalerate) (PHBV) with only minor effects on the thermal and mechanical properties. The results of this ecofriendly approach were validated by Ferri et al. [25]. The increase in the elastic modulus after adding wine lees to PHBV has been investigated [26]. Nanni et al. [27] further investigated the effects of wine lees and grape seed extracts on the mechanical and thermal properties of poly(3-hydroxybutyrate) (PHB). This study reported a reinforcement of PHB by wine lees and minor stabilization effects from grape seed extracts comparable to conventional antioxidants, such as Irganox 1010. In comparison, Persico et al. [2] observed increased thermal stabilization effects from WP extracts in PHB.

Another promising sustainable substitute for conventional plastics, besides the mentioned PLA and polyhydroxyalkanoates, is biobased poly(butylene succinate) (PBS). Its high ductility, comparable to PE, enables applications with injection-molded products and extruded films [28]. As for other polyesters, biobased PBS is susceptible to degradation mechanisms based on mechanical, thermal, and thermo-oxidative stresses [29,30,31]. Hallstein et al. [30] studied the degradation reactions of biobased PBS during processing. Thermo-oxidative degradation via radical reactions was found to be one of the main degradation mechanisms, especially under industrial conditions. By adding phenolic and phosphite antioxidants, branching reactions through thermo-oxidative reaction modes were prevented. Rizzarelli et al. [31] reported a significant reduction in the molar mass of PBS with thermo-oxidative degradation, confirming the need for antioxidative stabilizers for these types of polyesters. 

Several studies on the combination of biobased PBS and winemaking by-products have been published [32,33,34,35]. They either focused on the use of the by-products as non-functional fillers or on the use of extensively prepared extracts in PBS, as mentioned above. Gowman et al. [32] successfully incorporated WP in biobased PBS and observed an improvement in the mechanical properties, such as tensile strength and modulus, after adding a compatibilizer. Nanni et al. [33] reported an increase in the stiffness of PBS after adding dried and ground grape stalks as a reinforcing filler. Further, biocomposites based on PBS and wine lees showed improved mechanical properties and creep resistance with mostly unaltered thermal properties compared to neat PBS [34]. Regarding the stabilization of PBS by wine by-products, the effects of lab-made grape pomace extracts and commercial grape seed tannin extract were compared to the conventional stabilizer Irganox 1010 [35]. While the lab-made extract did not act as a stabilizer, the commercial extract prevented thermo-oxidative degradation comparably to the conventional stabilizer. These findings suggest that these by-products demonstrate high potential for use as antioxidant agents in biopolymers. Simultaneously, these results highlight the significant influence of the preparation methods on the efficiency of the bio-fillers and demonstrate the importance of appropriate processing parameters and material handling. 

Thermo-oxidative stabilization of PBS is not only crucial for processing but also for technical and long-term applications. Moreover, previous studies partly contradict the approach using a full valorization of the by-products. For these reasons, this research investigated the potential of WP as a whole for the improvement of the thermo-oxidative stability of biobased PBS. Additionally, in all previous studies, the wine by-products were dried and ground separately using non-industrial methods. The preparation methods could have a significant influence on the functional compounds, such as polyphenols, in the biobased fillers. Improper drying conditions and increased mechanical and thermal stresses during grinding can degrade the natural antioxidants [10]. Guaita et al. [7] identified drying at high temperatures for a short duration as the best strategy to prevent extensive degradation of phenolic compounds in WP. Nanni et al. [35] confirmed the need for appropriate by-product preparation to avoid degradation during handling and maintain the stabilization effects. In the present research, an industrial-scale method for WP preparation was used. WP was simultaneously dried and micronized with a TurboRotor mill-dryer, which is well-established in the food industry; e.g., for milling spices [36]. This method is characterized by high throughputs enabling upscaling [37] and its suitability for processing temperature-sensitive materials, such as phenolic compounds, due to its very short residence times and reduced thermal stress compared to conventional methods [38]. 

To the best of the knowledge of the authors, this is the first study to investigate the influence of industrial TurboRotor mill-dried WP on the thermo-oxidative stability of a biopolyester. Furthermore, this is the first study to characterize and use German WP as a functional filler, following a global approach with the principle transferable to different regions worldwide.

## 2. Materials and Methods

### 2.1. Materials and Chemicals

Commercially available biobased PBS (BioPBS FZ71PM) was purchased from PTT MCC Biochem Co., Ltd. (Bangkok, Thailand). The semi-crystalline polyester polymerized with biobased succinic acid and 1,4-butanediol has a density of 1.26 g/cm^3^, an MFR of 22 g/10 min (measured at 190 °C and 2.16 kg), and a melting temperature of 115 °C. The mechanical properties include a yield stress of 40 MPa, a stress at break of 30 MPa, and a strain at break of 170%. These data were taken from the material data sheet provided by the material supplier.

White WP from *Vitis vinifera* subsp. *vinifera* of the variety *Silvaner* (WWP-Silv), as well as red WP of the variety *Domina* (RWP-Dom), was kindly provided by the winery Richard Dahms GmbH (Schweinfurt, Germany) during harvesting in October 2021. White grapes were pressed immediately after harvest, while the red grapes had been previously macerated for 12 days. Both varieties were pressed on the same day. The fresh WP designated for processing was packed in mash barrels for transport and kept in cool storage until mill-drying. Native samples of WP without further preparation were stored in sealed bags at −18 °C until analysis. 

Bioethanol (99.9%) was supplied by Höfer Chemie GmbH (Kleinbittersdorf, Germany). All chemicals purchased for chemical analysis were analytical grade. Anhydrous sodium carbonate and 2,2′-azinobis-(3-ethylbenzothiazoline-6-sulfonic acid) (ABTS) were purchased from Applichem GmbH (Darmstadt, Germany). Folin–Ciocâlteu (FC) reagent was supplied by Merck KGaA (Darmstadt, Germany). Potassium persulfate, (±)-6-hydroxy-2,5,7,8-tetramethylchromane-2-carboxylic acid (Trolox), gallic acid, and 2,2-diphenyl-1-picrylhydrazyl (DPPH) were purchased from Sigma-Aldrich Chemie GmbH (Taufkirchen, Germany).

### 2.2. Preparation of Functional Bio-Fillers

Industrial mill-drying of the fresh WP using a TurboRotor mill was performed by Mahltechnik Görgens GmbH (Dormagen, Germany). The processing of the fresh WP to obtain dry and fine powders occurred in two stages. In the first stage, both WPs were processed with a rotor speed of 94 m/s at an air flow of 2000 kg/h and an elevated air intake temperature of 220 °C for simultaneous drying. The second stage for fine micronization was performed at a rotor speed of 113 m/s and an air flow of 1800 kg/h. In this stage, the air was not pre-heated to increase the intake temperature. These parameters were set by Mahltechnik Görgens based on their experience in processing biogenic materials. After milling, the micronized particles were separated from the air stream with a cyclone and manually bagged. The resulting dry bio-filler powders were stored in sealed bags in the absence of light to avoid degradation until processing and analysis.

### 2.3. Characterization of Native By-Products and Functional Bio-Fillers

#### 2.3.1. Characterization of Native Wine Grape Pomace

For both varieties, representative amounts of a minimum of 25 g WP were manually separated into their constituents (grape skins, seeds, and stalks) and weighed to obtain the initial mass on a fresh-weight basis (fwb). The separated constituents were dried in a dry-air T 5050 E laboratory oven (Heraeus Noblelight GmbH, Hanau, Germany) at a temperature of 105 °C for 48 h to ensure that all moisture was removed. After cooling in a desiccator to prevent moisture absorption from the atmosphere, the mass on a dry-weight basis (dwb) was determined. The relative moisture (RM) of the native by-products and their constituents was calculated using Equation (1):

(1) $$RM\;\%=\left(1-\frac{m_{dry}}{m_{fresh}}\right)\times 100$$

where $m_{fresh}$ and $m_{dry}$ represent the sample mass before and after the drying procedure, respectively. The shares on both fresh- and dry-weight bases were calculated as the ratio of the mass of the constituent and the total mass of the WP sample considering all constituents. The results are average values of two measurements.

#### 2.3.2. Characterization of Bio-Filler Powders

The particle size distribution of the prepared bio-filler powders was determined with a Cilas particle size analyzer 1090 (Cilas S.A., Orleans, France) in the range of 0.02–500.00 µm. A mixture of bio-filler powder, water, and 5 vol.-% tenside was prepared. Measurements were performed at an obscuration of 15% in triplicate. Results are expressed as the top cut $D_{97}$, mean and median particle size $\bar{D}$ and $D_{50}$, density, and cumulative distribution. 

The RM of the prepared WP powders was determined in duplicate with a KERN moisture analyzer DBS (Kern and Sohn GmbH, Balingen-Frommern, Germany). At least 5 g of powders was used. Measurements were performed in AUTO mode at a temperature of 105 °C using a preset weight loss ΔM of 0.05% as the shutoff criterion. Drying was completed when the weight loss remained constant (below the preset ΔM) for 30 s.

Thermal properties of the bio-filler powders were characterized using thermogravimetric analysis (TGA) with a Netzsch TG 209 F3 Tarsus (NETZSCH-Gerätebau GmbH, Selb, Germany). For each bio-filler, 9.5 ± 0.5 mg of powder was measured in a temperature range from 40 °C to 600 °C at a constant heating rate of 10 °C/min. The gas flows for purge and protective gas were set at a constant rate of 20 mL/min of nitrogen. Decomposition of the material was determined using the temperature at a mass loss of 5% ($T_{5\%}$), as well as the tangential onset temperature ($T_{on}$) at the maximum degradation rate obtained from the peak ($T_{p}$) of the first derivative of the TG signal (DTG).

#### 2.3.3. Chemical Analysis by Assays

Different spectrophotometric assays were carried out for chemical analysis of the by-products regarding their potential for use as stabilizing functional fillers. Prior to the spectrophotometric assays, liquid extracts of WP powders were produced via ultrasound-assisted extraction (UAE). Sample masses of 1 g were dissolved in 10 mL of extraction solvent (50/50 *v/v* ethanol/water) in a sealed container and well-agitated. The container was then sonicated for 1 h at 50 °C, a frequency of 40 kHz, and a power of 600 W in a Palssonic Eco UD30 ultrasound bath (Allpax GmbH and Co. KG, Papenburg, Germany). Sonication was briefly paused after 30 min to re-mix the samples. The liquid and solid phases of the samples were separated via centrifugation at 3500 rpm for 15 min in a Labofuge 200 (Heraeus Noblelight GmbH, Hanau, Germany). For each of the following assays, absorbance measurements were performed on a DR6000 UV-VIS spectrophotometer (Hach Lange GmbH, Düsseldorf, Germany). All assay measurements were performed in duplicate, and the results were calculated while considering the RM of the samples and averaged.

The FC assay, used to evaluate the total phenolic content (TPC), followed a procedure derived from Singleton et al. [39]. The previously obtained liquid extracts were diluted by a factor of 10 such that the absorbance was within a range covered by the gallic acid standard. A volume of 0.1 mL of the diluted liquid extract was mixed with 7.9 mL of deionized water and 0.5 mL of FC reagent. After an incubation time of 8 min, 1.5 mL of a 20% *w/v* aqueous sodium carbonate solution was added. The solutions were well-agitated and left to react in the dark for 30 min at 40 °C. Sample absorbance values were measured at 765 nm. A blank solution was studied using deionized water instead of liquid extract and subtracted from the absorbance values for all measurements. A calibration line using a gallic acid standard (y = 0.9568x; R^2^ = 0.9982) was generated. Results for each sample were compared to the gallic acid standard to determine the TPC of the sample in units of mg gallic acid equivalents (GAE) per g of WP dwb. 

The DPPH assay was performed using the procedure described by Brand-Williams et al. [40]. Six aliquots of the liquid sample extract were produced through dilution by a factor of 1–500, depending on the by-product, to obtain data points from 10 to 90% inhibition. A volume of 3.9 mL of a 60 µM DPPH radical cation solution in ethanol was mixed with 0.1 mL of the extract aliquot. The reaction proceeded in the dark for 30 min at room temperature (RT) before measurement. At 515 nm, the absorbance of each sample was measured. A blank solution (only ethanol) and a control solution (3.9 mL of DPPH solution and 0.1 mL ethanol) were also measured. The blank was subtracted from the absorbance value for each aliquot. The % inhibition of each aliquot was calculated using Equation (2):

(2) $$\%\;inhibition=\left(1-\frac{A_{S}}{A_{0}}\right)\times 100$$

with $A_{S}$ representing the absorbance of the sample and $A_{0}$ representing the absorbance of the control (where no DPPH was inhibited). From this, a curve representing the % *inhibition* against the concentration of WP (dwb) in the extract was obtained. The value for inhibition of 50% of the DPPH ($IC_{50}$) was determined for each sample using linear regression analysis. The $IC_{50}$ of the sample was compared to that derived from a Trolox standard curve (y = 497.5701x; R^2^ = 0.9985) to obtain the radical scavenging activity in units of mmol Trolox equivalents (TE) per 100 g dwb.

The ABTS assay method was adapted from Re et al. [41] with minor modifications. An ABTS radical solution was prepared by mixing a 7 mM aqueous ABTS solution with a 140 mM aqueous potassium persulfate solution to obtain a final concentration of 2.45 mM. The solution was left to activate at RT in the dark for 16 h before use. Subsequently, the solution was diluted with ethanol until it adopted an absorbance of 0.700 ± 0.005 at a wavelength of 734 nm. For each sample studied, six aliquots were prepared by diluting the liquid extract by a factor of 1 to 500 depending on the by-product studied. A volume of 3.4 mL of the ABTS solution was mixed with 0.1 mL of the aliquot. The reaction was allowed to proceed for 6 min at RT before its absorbance at 734 nm was measured. A blank sample consisting of 100% ethanol and a control sample using 3.4 mL ABTS working solution and 0.1 mL ethanol were also measured. The blank was subtracted from the sample absorbances. Using the control sample, a % inhibition versus sample concentration curve was generated using Equation (2). The $IC_{50}$ value for the curve was determined through linear regression analysis and compared to that obtained from a Trolox standard curve (y = 456.3230x; R^2^ = 0.9995). Values for the antioxidant capacity are expressed in units of mmol TE per 100 g dwb sample.

### 2.4. Preparation of Biocomposites Based on PBS and Bio-Fillers

Biocomposites based on PBS and WP bio-fillers were compounded with a Labtech type LTE20-44 laboratory co-rotating twin-screw extruder with an L/D ratio of 44 and screw diameters of 20 mm (Labtech Engineering Co., Ltd., Samutprakarn, Thailand). Prior to processing, the matrix material was pre-dried according to the manufacturer recommendations for at least 5 h at 80 °C in a BIN S 15 in combination with a LUXOR 50 dry air generator (Motan Holding GmbH, Konstanz, Germany). Filler concentrations of 0, 5, 10, 15, and 20 wt.-% were compounded. The fillers were incorporated using a Labtech type LSF20-10 side feeder controlled by a gravimetric dosing system (Scholz Dosiertechnik GmbH, Großostheim, Germany). The temperature profile for compounding is reported in Table 1. The screw speed was set at 260 rpm for all recipes at throughputs of 6.0–6.5 kg/h. The melt-mixed materials were cooled in a water bath and granulated with a Labtech type LZ-120/hp granulator for subsequent processing and analysis. The compounds were stored in sealed bags in the absence of light until subsequent processing and/or analysis.

Tensile test specimens (type four according to the technical standard DIN EN ISO 8256) were injection-molded using a BOY XS injection-molding machine (BOY Machines, Inc., Exton, PA, USA). Before injection molding, the compounds were pre-dried for 5 h at 80 °C, similar to the pre-drying for compounding. Injection molding was performed at temperatures of 155 °C, 160 °C, and 170 °C from hopper to nozzle and with a mold temperature of 25 °C. The injection pressure of 800 bar maximum, the injection speed of 3 cm^3^/s, the holding pressure of 600 bar maximum, the holding time of 2 s, and the cooling time of 20 s were kept constant throughout the injection molding of all test specimens. 

### 2.5. Characterization of Biocomposites

#### 2.5.1. Thermal Properties

Thermal properties of the composites were determined using differential scanning calorimetry (DSC) on a Netzsch DSC Polyma 214 system (NETZSCH-Gerätebau GmbH, Selb, Germany). A sample mass of 15 ± 1 mg and gas flows of 40 mL/min (purge) and 60 mL/min (protective) of nitrogen were used. The program consisted of a first heating cycle from −60 °C to 180 °C, followed by a cooling cycle from 180 °C to −60 °C and a second heating cycle from −60 °C to 180 °C. Each cycle was performed at a constant heating rate of 20 °C/min and separated from the others by isothermal segments of 3 min. The melting peak temperature ($T_{m}$) and the melting enthalpy ($∆H_{m}$) were determined from the second heating cycle, whereas the crystallization peak temperature ($T_{c}$) and the crystallization enthalpy ($∆H_{c}$) were obtained from the cooling cycle. Thereby, the degree of crystallinity (${Χ}_{c}$) was calculated considering the weight fractions (w) of the filler and the theoretical melting enthalpy of 100% crystalline PBS ($∆H_{m}^{0}$) of 200 J/g [32,42], following Equation (3):

(3) $${Χ}_{C}\;\%=\frac{∆H_{c}}{∆H_{m}^{0}\left(1-w\right)}\times 100$$

As for the measurements of the bio-filler powders, a Netzsch TG 209 F3 Tarsus (NETZSCH-Gerätebau GmbH, Selb, Germany) was used for TGA. A sample mass of 9.5 ± 0.5 mg was heated from 40 °C to 600 °C at a constant heating rate of 10 °C/min. During heating, the chamber was purged with gas flows of 20 mL/min of nitrogen for both purge and protective gas. Initial decomposition was obtained from the temperature ($T_{10\%}$) at a mass loss of 10%, while the onset temperature (*T_on_*) was determined using a tangential procedure at the maximum degradation rate obtained from the DTG curve. Both the DSC and the TGA measurements were performed in duplicate, and the results are expressed as mean values.

#### 2.5.2. Thermo-Oxidative Properties

To determine the thermo-oxidative stability of the compounds, dynamic oxidation induction temperature (OIT) measurements were conducted on a Netzsch DSC Polyma 214 (NETZSCH-Gerätebau GmbH, Selb, Germany). An open aluminum crucible with a sample mass of 5.0 ± 0.5 mg was used. The measurement cell was purged with oxygen for 5 min at 25 °C at a flow rate of 50 mL/min (protective: 60 mL/min nitrogen). Subsequently, the oven was heated from 25 °C to 330 °C at a constant heating rate of 20 °C/min. Degradation temperatures were determined using the offset method with a Δ of 0.05 W/g ($OIT_{off}$) and the tangential onset procedure at the maxima of the first derivative of the curve according to the technical standard DIN EN ISO 11357-6. Since PBS shows two-step oxidative degradation, both maxima of the first derivative of the curve were considered for onset temperature determination ($OIT_{on1}$, $OIT_{on2}$). All OIT measurements were performed in triplicate.

As a second method to evaluate the thermo-oxidative properties of the materials, TGA was performed in an oxidative atmosphere. Due to the design of the Netzsch TG 209 F3 Tarsus (NETZSCH-Gerätebau GmbH, Selb, Germany), a maximum oxygen concentration of 50% was achievable at a total gas flow of 40 mL/min (purge: 20 mL/min oxygen, protective: 20 mL/min nitrogen). The values ox. $T_{10\%}$, ox. $T_{on}$, and ox. $T_{p}$ (obtained from the DTG) were determined in duplicate in the same manner as the TGA measurements performed in a nitrogen atmosphere.

#### 2.5.3. Mechanical Properties

Mechanical properties of neat and filled PBS were characterized via tensile testing of the injection-molded specimen. A Zwick RetroLine Z 2.5 (ZwickRoell GmbH and Co. KG, Ulm, Germany) equipped with a 2.5 kN Xforce P load cell was used. Testing was performed at ambient conditions with a clamp length of 30 mm and 1 mm/min clamp separation speed for determination of the tensile modulus ($E_{t}$) and 50 mm/min for the tensile strength ($\sigma_{M}$) and the elongation at break ($\varepsilon_{b}$), respectively. All mechanical data are expressed as the average for at least five specimens, which were previously stored in a desiccator for at least 48 h.

## 3. Results and Discussion

### 3.1. Composition of Native Wine Grape Pomace

As a result of destemming during harvesting, WP obtained after pressing mainly consists of grape skins and seeds. Nevertheless, residual stems still account for 3–5% of WP [21]. As shown in Table 2, there were minor differences in composition between the WWP-Silv and the RWP-Dom. The variations concerned the share of residual stems on both fresh- and dry-weight bases, which affected the shares of the remaining constituents. While RWP-Dom had a share of stems of 4.88% (dwb), WWP-Silv only contained about half the amount of stems (2.24%). While the share (fwb) and RM of the seeds were almost equal in both WP varieties, the stems and skins of WWP-Silv were higher in moisture, leading to an elevation of nearly 3.4% in total RM compared to RWP-Dom. 

The fact that the difference in the share of stems was the only significant variation (ANOVA; *p* = 0.05) confirms that the compositions of both WP were comparable. Although the total RM of WP in this study tended to be lower than those of some WPs investigated in previous studies [10,15], it was generally consistent with the range of 50–72% reported in the literature [12,43,44]. The values for the present WWP-Silv were comparable to results for white varieties, such as *Chardonnay*, *Macabeu*, and *Paellada*, cultivated on Mallorca Island in Spain [44] and other varieties cultivated in Virginia (USA) [43]. The RMs of the whole WP and the stems of the RWP-Dom were also comparable to red varieties cultivated in Spain and Virginia investigated in previous studies [43,44]. Regarding the shares of the different constituents, the German WPs were 20–30% higher in seeds (fwb) compared to WPs from the US investigated by Jiang et al. [45]. In the literature, the general ratio of skins to seeds in WP is reported to be roughly 1:1 [5,21,46]. On a dwb, for both varieties of WP, the proportion of seeds was slightly higher than the share of skins. Remarkably, WWP-Silv was almost 4% richer in seeds on a dwb than RWP-Dom. However, these results confirm that the compositions of by-products from different regions are generally comparable, apart from minor differences relating to grape variety, cultivation area, and winemaking processing parameters [5,47].

### 3.2. Characterization of Bio-Filler Powders

#### 3.2.1. Physical Properties of Prepared WP Fillers

**Figure 2:**
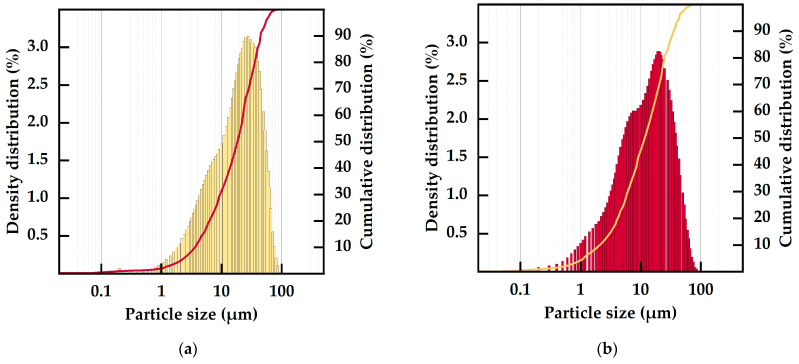
Particle size distribution (density and cumulative distribution) of (**a**) prepared WWP-Silv bio-filler powder and (**b**) prepared RWP-Dom bio-filler powder.

Fresh WPs were mill-dried using a TurboRotor mill-dryer as described above to obtain micronized and dry bio-filler powders. The particle size distributions of both micronized bio-fillers are displayed in Figure 2. As shown, micronization by the TurboRotor resulted in relatively narrow distributions of small particle sizes below 100 µm compared to other preparation methods [48]. Besides the main fraction of particle sizes in the range of 10–40 µm, both fillers consisted of finer particles with diameters of less than 10 µm, recognizable by the shoulder on the left side of the major density distribution peak. David et al. [48] observed a similar partial separation of grinded WP. This effect can be ascribed to differences in the grindability of the complex lignocellulosic structures of the by-products, as reported for the centrifugal grinding of wheat straw [49]. The share of the fraction 1–10 µm in size was greater for RWP-Dom than for WWP-Silv. In addition, the WP powders of both varieties contained minor shares of nanoscale particles. Although both WPs were mill-dried using identical parameters, slight differences in the particle size distributions were observed. These variations may have been related to the higher initial RM and the composition of the fresh WWP-Silv. Grinding of materials with increased RM results in larger average particle sizes due to the plasticizing effect of moisture [50,51]. As displayed in Table 2, there were slight differences in the initial compositions of the fresh WP, which may have additionally led to the particle size distribution being slightly shifted to increased particle sizes.

In total, the particles of RWP-Dom were smaller in size compared to WWP-Silv, leading to top cuts $D_{97}$ of 51.97 µm (RWP-Dom) and 62.37 µm (WWP-Silv), as reported in Table 3. This trend was also consistent for the median particle size $D_{50}$, with RWP-Dom particles being 34.45% smaller in size, as well as the mean diameter, with a difference of 27.27%. The top cut and the particle size distribution of the fillers do not only influence the mechanical properties of the biocomposites [52] but also the efficiency of the functional fillers stabilizing the matrix polymer [53,54,55]. Additionally, small-sized filler particles may act as nucleating agents, affecting the crystallinity of the matrix polymer [34]. Therefore, the present differences in particle size may have affected the chemical and mechanical properties of the resulting biocomposites.

In terms of drying, the micronized powder of WWP-Silv had about 0.7% higher RM (absolute) compared to RWP-Dom (Table 3). Since both varieties were processed identically, the increased moisture content of the WWP can be attributed to the approximately 3.4% higher initial moisture of the fresh WP previously mentioned in Table 2. Sant’Anna et al. [56] found Brazilian WP powder of the variety *Labrusca* with a maximum moisture content of 9.7% to be microbiologically stable for storage at ambient conditions. Nevertheless, the RMs of both micronized fillers in this study were below 5%, which is considered the limit for safe storage of dairy ingredients [57,58]. These results confirm mill-drying with a TurboRotor as a viable preparation method to obtain fine biogenic powders with suitable moisture contents in addition to economic benefits. 

#### 3.2.2. Thermal Properties of Prepared WP Fillers

**Figure 3:**
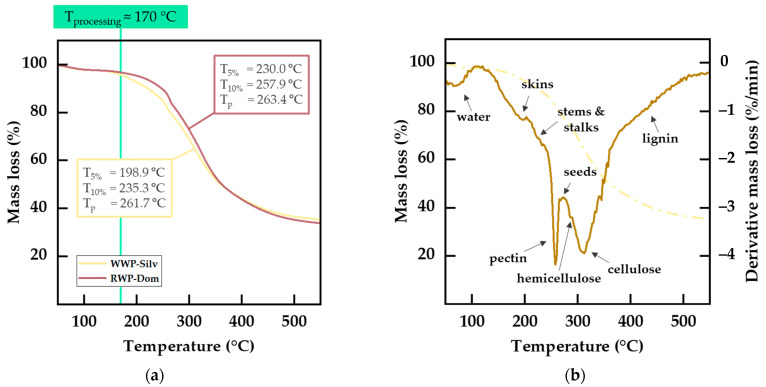
Thermal properties of prepared WP fillers: (**a**) thermogravimetric (TGA) mass loss curves for WWP-Silv and RWP-Dom, including determined degradation temperatures with respect to the processing temperature of the PBS matrix; (**b**) derivative mass loss (DTG) curve for WWP-Silv and characterization of WP constituents.

The thermal stability of WP powders is of great importance for their use as functional fillers in polymer materials. During compounding, the biogenic fillers are exposed to the processing temperatures of the thermoplastic matrix and shear stress. Hence, the intrinsic thermal stability of the fillers must exceed these temperatures to avoid premature degradation and further reactions by the degradation products. Figure 3a displays the mass loss curves of WP fillers measured using TGA in a nitrogen atmosphere. Both fillers showed smooth degradation in multiple steps, with slightly higher thermal stability for RWP-Dom compared to WWP-Silv. The initial thermal degradation was characterized by a $T_{5\%}$ of 198.9 °C (WWP-Silv) and 230.0 °C (RWP-Dom), as reported in Table 4. Thus, both WPs were thermally stable and suitable to be embedded as functional fillers in the PBS matrix with processing temperatures of approximately 170 °C. 

By analyzing the DTG curve, additional information on the thermal stability of the WP ingredients can be obtained. In Figure 3b, the DTG curve for WWP-Silv is shown. Since WP is a lignocellulosic biomass, hemicellulose, cellulose, and lignin are found with ascending thermal stability in this order [59]. In the literature, the decomposition of hemicellulose is reported to occur in the range of 200–350 °C, while cellulose usually degrades at 280–350 °C and lignin decomposes at higher temperatures of up to 600 °C [4,48,59,60,61]. In contrast to other biomasses, WP exhibits a sharp peak at a temperature of about 260 °C. This additional peak can be related to pectin and non-structural sugars present in WP [4], limiting the overall thermal stability of the biogenic filler. Besides the general constituents of biomass, the typical constituents of WP can be identified from their characteristic decomposition areas. Grape skins are reported to have the lowest thermal stability, resulting in degradation temperatures of 180–195 °C, followed by stems, which decompose at 208–240 °C [11,21]. Degradation of grape seeds usually occurs at temperatures identical to the decomposition temperatures of prominent polyphenols, such as tannins and gallic acid (approximately 267 °C) [11]. With respect to the corresponding temperature ranges, RWP-Dom was about 1.3% richer in pectin and contained approximately 3.6% more cellulose.

#### 3.2.3. Chemical Properties of Prepared WP Fillers

The chemical properties of the prepared WP powders in terms of total phenolic content (TPC) and antioxidant activity (AA) were investigated to study their potential as stabilizers rather than solely as low-cost fillers. Assay results are reported in Table 5. The TPC of WWP-Silv powder was 71.76 mg GAE/g dwb WP. With a value of 74.73 mg GAE/g dwb, the TPC of RWP-Dom was 4.14% higher in polyphenolic compounds. The higher TPC in RWP-Dom was likely due to the higher proportion of anthocyanins, polyphenolic pigments responsible for the red color of the grape skins [5]. This is consistent with previous studies reporting higher TPC for red WP in the range of 16.60–51.02 mg GAE/g dwb [62,63,64,65] compared to 4.55–31.13 mg GAE/g dwb [66,67] for white varieties. The variations in TPC values are caused by a combination of factors, including wine variety, cultivation and processing methods, and assay techniques. Both present TPC values exceeded the range of values reported in the literature. This may have been a result of the gentleness of mill-drying, which limits degradation of polyphenolic compounds during processing [37]. The use of UAE, a more efficient extraction technique, could have led to the acquisition of a higher concentration of polyphenols in the liquid extract compared to traditional solid–liquid extraction methods with similar temperature, duration, and solvent parameters [68]. Additionally, the smaller particle sizes achieved during grinding and the resulting increase in surface area may have led to higher TPC values due to higher concentrations of polyphenols in the liquid extract [53,54].

The AA of the prepared WP powders was measured using DPPH and ABTS assays. Both assays quantify the antioxidant activity of hydrogen-donating antioxidants, while the ABTS assay also extends to chain-breaking antioxidants [40,41,69]. In combination, they can provide a comprehensive overview of the antioxidative character of bio-fillers. The DPPH assay yielded a radical scavenging activity of 51.32 mmol TE/100 g dwb for RWP-Dom, representing a value 15.46% larger than the 44.45 mmol TE/100 g dwb radical scavenging activity of WWP-Silv. When compared to the literature, the present values exceeded or fell on the higher end of the previously reported values. The DPPH assay usually yields values for red and white WP of 11–51 mmol TE/100 g dwb [70,71,72] and 22–36 mmol TE/100 g dwb [73,74], respectively. According to the ABTS assay, RWP-Dom had a Trolox-equivalent antioxidant capacity of 60.31 mmol TE/100 g dwb, 8.51% greater than the 55.58 mmol TE/100 g dwb obtained for WWP-Silv. In the literature, the ABTS assay yielded values for red and white WP of 15–60 mmol TE/100 g dwb [70,71,75,76] and 23–59 mmol TE/100 g dwb [44,63,72,77,78], respectively. The present values for the ABTS assay fell on the upper end of these reported regions. In both assays, the RWP-Dom outperformed WWP-Silv, likely due to its smaller particle size, which allows for more efficient extraction of polyphenolic compounds, and higher content of active compounds, such as pectin, as described in Table 4 [79,80]. Despite having higher TPC values than indicated in the literature, the DPPH and ABTS values tended to be within expected ranges. This was likely because UAE allows for the extraction of additional phenolic compounds that do not enhance the antioxidant properties of samples.

### 3.3. Thermal Properties of WP-Based Biocomposites

The results of the analysis of the thermal properties of WP-based biocomposites are reported in Table 6. Even at higher filler contents, neither of the WPs led to any significant changes in the characteristic thermal properties of the materials with respect to the melting behavior and the degree of crystallinity. The melting peak temperature ($T_{m}$) remained almost unchanged. These results are consistent with the findings of Gowman et al. [32] using Canadian WP. Similarly, the crystallization peak temperature ($T_{c}$) remained almost constant. Thus, neither $T_{m}$ nor $T_{c}$ were substantially affected by the fillers, as previously reported in the literature for various wine fillers [21,33]. 

${Χ}_{c}$ increased by only 0.3–1.6% at higher WWP-Silv filler contents and remained almost constant for RWP-Dom. Consequently, as with grape stalks, neither of the WPs acted as a pronounced nucleating agent [33]. In contrast, Nanni et al. [34] reported a strong nucleating effect for wine lees in PBS manifesting as an increase in the degree of crystallinity of 17.7% at a filler content of 20 phr. The nucleating effect was ascribed to the small mean diameter of the particles of about 25 µm. Although the mean diameters of the WP fillers were comparable in size or even smaller, only mild increases in the degree of crystallinity were observed. A possible reason for this could have been the different particle forms of the fillers [21,27]. For an increased degree of crystallinity, compatibilizers could be added to increase matrix–filler interaction [32].

**Figure 4:**
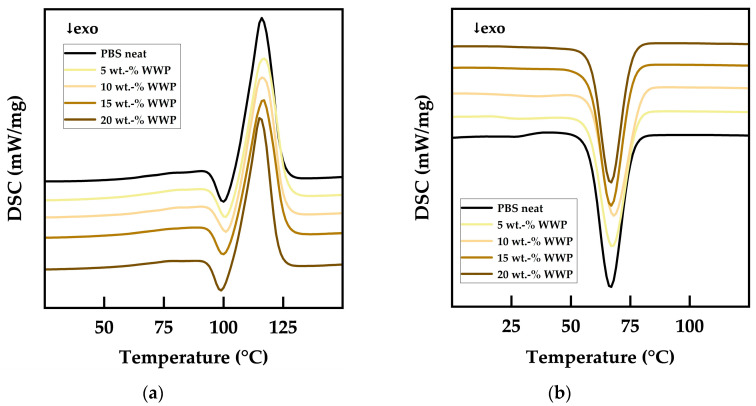
Stacked DSC curves for neat PBS and WWP-Silv biocomposites: (**a**) second heating scan; (**b**) cooling scan.

No readjustments were required for stable processing of the materials since the thermal behavior of the composites was comparable to that of neat PBS even at higher filler contents for both WPs. This observation was confirmed by DSC analysis, as demonstrated by the stacked DSC curves of the melting process from the second heating scan in Figure 4a and the stacked graphs of the crystallization from the cooling scan in Figure 4b. All samples showed similar graphs, almost congruent, with no additional peaks or peak separations, proving that the thermal properties of PBS were not significantly affected by WP fillers.

The thermal stability of the biocomposites was determined using TGA measurements in a nitrogen atmosphere to exclude oxidative degradation processes. The values obtained are listed in Table 7. While neat PBS showed an initial decomposition temperature of 362 °C ($T_{10\%}$) and a $T_{on}$ of about 370 °C with a peak in the DTG curve ($T_{p}$) at 400 °C, all temperatures were lowered with increasing filler content. With losses of 4% (WWP-Silv) and 6% (RWP-Dom), the highest reduction in the $T_{10\%}$ was observed for PBS filled with 20 wt.-% WP. $T_{on}$ was reduced by 1–3%, depending on the filler content, as the DTG peak temperatures decreased. This phenomenon was reported in previous studies using wine by-products as fillers in biopolymers and can be explained by the lower intrinsic thermal stability of the biogenic fillers compared to the matrix material [21,32,33]. As a result, the overall thermal stability of the biocomposites was reduced in accordance with the WP filler content.

**Figure 5:**
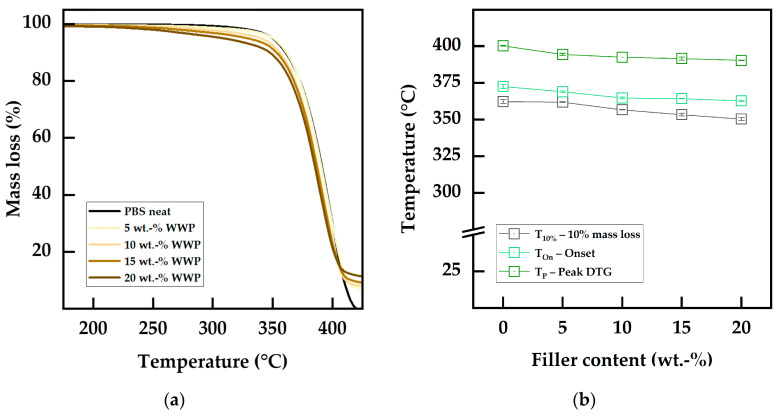
Thermal stability of neat PBS and WWP-Silv biocomposites: (**a**) TGA mass loss curves measured in a nitrogen atmosphere; (**b**) degradation temperatures as a function of the filler content.

In Figure 5a, the shift of the mass loss curves of the biocomposites towards lower temperatures can be observed. By comparing them with the mass loss curve of pure WP shown in Figure 3a, it can be seen that the curve assumed the shape of the filler-only curve with increasing filler content. The mass losses at temperatures below 300–350 °C can be ascribed to the less thermally stable WP constituents, including pectin and hemicellulose [4]. While these compounds tend to degrade at lower temperatures than the matrix, the lignin in WP was responsible for the increased remaining mass at temperatures above 400 °C [61]. However, despite the earlier degradation plotted in Figure 5b, the thermal degradation occurred at comparable temperatures and was characterized by a single step for both the filled and the neat PBS [4]. The ability of the biocomposites to withstand thermal degradation mechanisms was still comparable to neat PBS up to the highest filler contents of 20 wt.-%. Consequently, these results show that WP did not act as a thermal stabilizer to avoid the pure thermal degradation of PBS through β-hydrogen-transfer bond scission [31,81].

### 3.4. Thermo-Oxidative Properties of WP-Based Biocomposites

Previous studies have stated that thermo-oxidative mechanisms are the main degradation phenomena for PBS, especially during processing [30,31,81]. For a holistic characterization of the oxidative degradation, initial radical reactions were detected with the offset method ($OIT_{Off}$) and the onset temperatures of the proceeding oxidation were determined tangentially from the maxima of the first deviation ($OIT_{On}$), as reported in Table 8. Starting with the neat PBS sample, besides the initial radical reactions occurring at 216.6 °C ($OIT_{Off}$), two-staged degradation was observed at approximately 246 °C ($OIT_{On1}$) and 278 °C ($OIT_{On2}$). This thermo-oxidative, stress-dependent, multistep degradation can be related to the different degradation pathways suggested by Rizzarelli et al. [31]. Thermo-oxidative decomposition of PBS starts with an α-hydrogen abstraction mechanism and the formation of unstable hydroperoxide intermediates. Further reactions proceeding through the elimination of hydroxyl radicals lead to the formation of macroradicals. Macroradical formation is followed by three different degradation pathways depending on the load of thermo-oxidative stress, resulting in oligomers with different end-groups [31].

**Figure 6:**
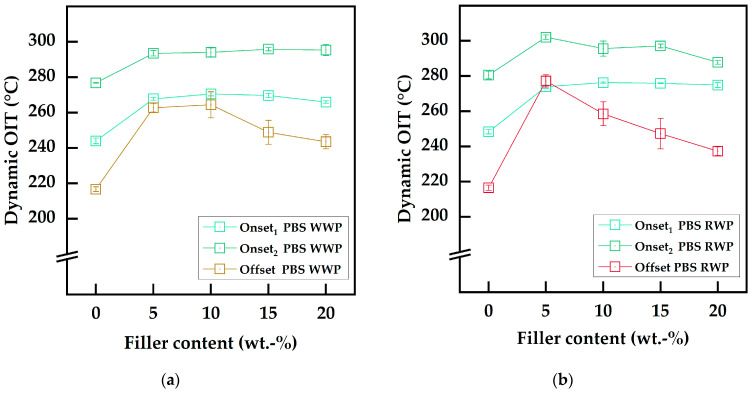
Thermo-oxidative degradation temperatures determined using the dynamic OIT as a function of the filler content: (**a**) neat PBS and WWP-Silv composites; (**b**) neat PBS and RWP-Dom composites.

The incorporation of WP fillers led to increased thermo-oxidative stability in the biocomposites compared to neat PBS. Natural polyphenols, as contained in WP, are radical scavengers capable of deactivating initially formed radicals, which suppress radical chain reaction degradation processes [82]. As a result, a remarkable elevation in the $OIT_{Off}$ was observed with maximum temperatures at lower filler contents. WWP-Silv led to an increase of 46.0 °C at 5 wt.-%, with an optimum at 10 wt.-% filler content (+47.7 °C). At higher filler contents, the $OIT_{Off}$ decreased to a value of 243.5 °C but still outperformed the neat PBS by 26.8 °C. Concerning RWP-Dom, maximum stabilization was reached at 5 wt.-% with an $OIT_{Off}$ of 277.2 °C (+60.7 °C). With increasing filler content, the initial degradation temperature decreased to 237.3 °C (20 wt.-%). These optima for thermo-oxidative stabilization regarding the initial radical reaction are visualized in the graphs in Figure 6. The maximum for the RWP-Dom biocomposites was reached at lower filler contents compared to the WWP-Silv-based counterparts. This was consistent with the results from the chemical filler characterization determined using spectrophotometric assays, where higher radical scavenging and antioxidant activities for RWP-Dom were observed. This indicates that RWP-Dom is a more efficient thermo-oxidative stabilizer. The optimum concentrations can be ascribed to pro-oxidation effects, often reported for antioxidant agents and natural additives [83,84,85]. Shares of stabilizers greater than the optimum concentration may support the formation of reaction products, leading to undesirable side reactions. This is a phenomenon also observed for conventional stabilizers, such as Irganox 1010 [86].

In contrast to the $OIT_{Off}$, the increment in the onset temperatures for the two-staged thermo-oxidation from the incorporation of the WP fillers did not show comparable maxima. Instead, the fillers led to the formation of a plateau at around 270 °C ($OIT_{On1}$) and at approximately 300 °C ($OIT_{On2}$). The fact that RWP-Dom outperformed WWP-Silv was also evident in the onset temperatures. While WWP-Silv biocomposites showed $OIT_{On1}$ values of 265.9–270.5 °C, temperatures of 273.9–276.2 °C were obtained for composites based on RWP-Dom. Slightly higher $OIT_{On1}$ values were reached at 10 wt.-% for both WPs, resulting in increases of 26.6 °C (WWP-Silv) and 27.9 °C (RWP-Dom) compared to neat PBS. $OIT_{On2}$ values of 293.4–295.3 °C (WWP-Silv) and 287.7–302.1 °C (RWP-Dom) highlight that the stabilization effects with higher-intensity thermo-oxidative stress were comparable for both functional WP fillers. Here, the stability increased by about 20 °C. These results confirm that the natural antioxidants in WP are able to suppress radical-based chain reactions during thermo-oxidative degradation by inactivating free radicals due to the radical scavenging activity of the polyphenols [23,82,83]. Intermediates with high reactivity were also stabilized, as seen by the higher onset temperatures. Consequently, the matrix material was stabilized, and degradations were shifted to higher temperatures.

**Figure 7:**
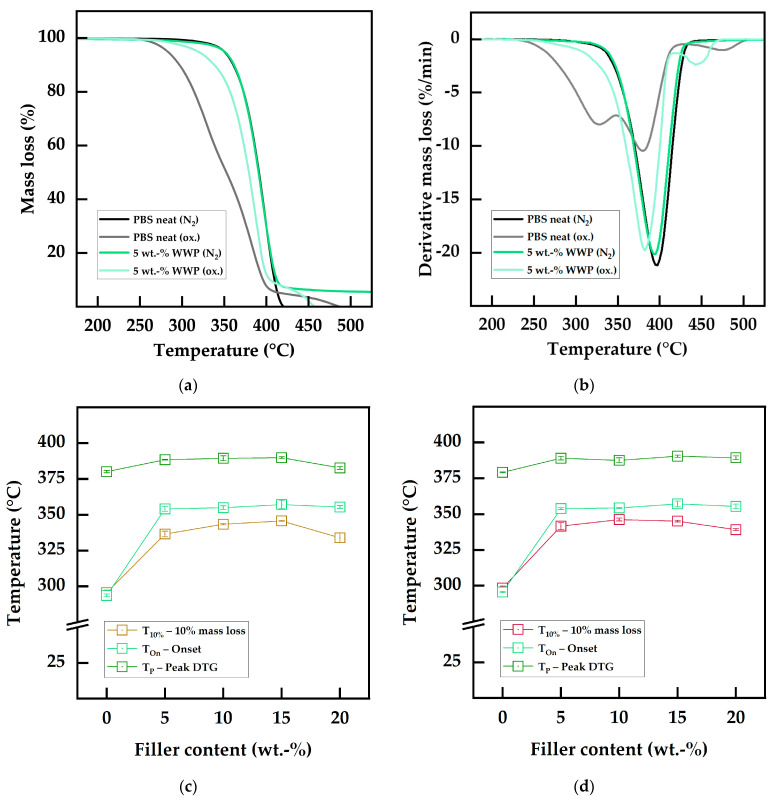
Thermo-oxidative properties determined using oxidative TGA: (**a**) TGA curves of neat PBS and biocomposites filled with 5 wt.-% WWP-Silv in nitrogen and oxidative atmospheres; (**b**) corresponding DTG curves. Thermo-oxidative degradation temperatures determined using oxidative TGA as a function of the filler content: (**c**) neat PBS and WWP-Silv composites; (**d**) neat PBS and RWP-Dom composites.

In Figure 7a, the TGA mass loss curves for neat PBS and PBS composite filled with 5 wt.-% WWP-Silv in nitrogen and oxidative atmospheres are compared. While the curves for both materials were almost congruent in the nitrogen atmosphere due to the similar thermal properties described above, distinct differences were visible for the measurements in the oxidative atmosphere. In the presence of oxygen, neat PBS showed two-staged decomposition with earlier degradation at remarkably lower temperatures through the occurrence of oxidative reactions. In contrast, the degradation of the biocomposite filled with 5 wt.-% occurred in one major decomposition step. The character of the degradation remained comparable with that in a nitrogen atmosphere, with a shift to lower temperatures by about 10 °C. Further details on the decomposition processes can be obtained from the derivative mass loss curve displayed in Figure 7b. In a nitrogen atmosphere, the filled and neat PBS showed a single degradation peak slightly below 400 °C, which is attributed to the aforementioned thermal decomposition of the material [32,35]. In an oxidative atmosphere, neat PBS showed three decomposition stages. The second peak, which occurred at comparable temperatures and had the greatest intensity, primarily represents thermal decomposition, as in the nitrogen atmosphere. A third decomposition stage with the least mass loss occurred at 480 °C and can be associated with the oxidation of residues from previous degradative reactions [87]. The additional first peak around 330 °C can be ascribed to thermo-oxidative degradation [35]. Remarkably, this peak almost completely disappeared when WP was added to the PBS matrix, indicating the antioxidant character of the filler. Thermo-oxidative degradation mechanisms were suppressed such that thermal degradation occurred almost exclusively, as reported for conventional stabilizers and natural extracts by Nanni et al. [35].

Accordingly, the increased thermo-oxidative resistance was indicated by the values reported in Table 9. Compared to neat PBS with degradation temperatures $T_{10\%}$ and $T_{on}$ below 300 °C, all biocomposites showed higher stability, leading to temperatures above 330 °C. The increase of approximately 40–50 °C indicated improved performance for the WP functional fillers when compared to conventional stabilizers, such as Irganox 1010 and commercially or extensively prepared extracts at concentrations of 1 phr [35]. Interestingly, in contrast to the OIT results, the highest values were observed for WP-based biocomposites at higher filler contents of 10–15 wt.-%. Since the mass loss of a sample as a consequence of volatile decomposition products is detected as decomposition proceeds, these degradation temperatures are generally higher than the OIT temperatures. The determined thermo-oxidative degradation temperatures versus the filler content are displayed in Figure 7c,d (WWP-Silv biocomposites and RWP-Dom biocomposites, respectively). The trend of RWP-Dom outperforming WWP-Silv, as discussed regarding the OIT data, was consistent with the results of the oxidative TGA. Further, the oxidative $T_{on}$ and $T_{p}$ for the composites were around 355 °C and 390 °C, with no major influence from the filler content. As reported in the literature, due to the successful limitation of the oxidative reactions, these values can be mainly ascribed to pure thermal degradation of the material, explaining their uniformity [35]. As displayed in Figure 7c,d, the graphs of $T_{10\%}$ and $T_{on}$ were very similar, almost congruent, to those for the $OIT_{On1}$ and $OIT_{On2}$ from the dynamic OIT analysis (Figure 6a,b). Overall, the stabilizing effects of WP functional fillers against thermo-oxidative degradation mechanisms observed in the OIT analysis were confirmed by the oxidative TGA measurements.

### 3.5. Mechanical Properties of WP-Based Biocomposites

In contrast to conventional polymer additives, the functional fillers were incorporated in comparatively high concentrations of up to 20 wt.-%. Accordingly, potential changes in the mechanical properties of the biocomposites had to be investigated to determine the influence of the WP fillers. The mechanical properties (tensile modulus (*E_t_*), tensile strength ($\sigma_{M}$), and elongation at break ($\varepsilon_{b}$)) of the neat PBS and biocomposites based on WP fillers are reported in Table 10.

As previously reported in the literature, incorporation of WP leads to an increase in the tensile modulus with increasing filler content [21,32]. The correlation of *E_t_* and filler content was linear in nature, with R^2^ = 0.9978 for WWP-Silv and R^2^ = 0.9923 for RWP-Dom. The maximum $E_{t}$ was reached at the highest filler contents of 20 wt.-% with 1257 MPa (WWP-Silv) and 1403 MPa (RWP-Dom). Since the degree of crystallinity of the PBS matrix remained almost constant, the reason for the increase in the tensile modulus of the biocomposites can be ascribed to the incorporated fillers. With the addition of fillers, macromolecular chain mobility was reduced, leading to increased deformation resistance [32,88]. Further, $E_{t}$ was increased by the higher intrinsic stiffness of WP resulting from its lignocellulosic constitution [21,33]. Compared to the stiffening effect of grape stalks in PBS, which showed an increase of 17–18%, WP was not as reinforcing, leading to smaller gains of 9% (WWP-Silv) and 12% (RWP-Dom) at comparable concentrations. According to Nanni et al. [21,33], this difference is a consequence of grape stalks being richer in cellulose than WP, providing a higher rigidity than other lignocellulosic compounds, such as hemicellulose and lignin. This is confirmed by the findings of Dominguez et al. [89], where lignin did not substantially increase the $E_{t}$ of PBS. In addition to higher stiffness, relative humidity has a lower impact on cellulose than other hemicellulose and lignin compounds [90]. Biocomposites based on RWP-Dom were slightly more rigid than the WWP-Silv-based counterparts. This was expected due to the higher share of stalks in RWP-Dom, which provided increased rigidity due to the cellulose contained (Table 4). Marcuello et al. [91] found larger interfacial interactions between PBS and cellulose, further supporting the stiffening effects. Additionally, higher $E_{t}$ may be achieved through better particle dispersion and homogeneity, as reported for wine lees fillers, since the RWP-Dom powder particles were smaller in size, as shown in Table 3 [26,34].

On the other hand, the tensile strength was reduced from the above to values below 40 MPa, with a further decrease to approximately 29 MPa for filler contents of 20 wt.-%. Gowman et al. [32] reported a drop in tensile strength by 60% for materials filled with 50 wt.-% Canadian WP due to poor interaction between the WP and PBS. Comparable results were obtained for German WP in this study, indicating low interfacial interaction and particle–matrix adhesion [21,32]. Accordingly, mechanical stress could not be transferred through the interface and the formation of crazes and crack propagation were facilitated. An increase in tensile strength from a decrease in the filler particle size, as reported in the literature [21,52], was not clearly observed in this study. The difference in the particle sizes of WWP-Silv and RWP-Dom might have been too small to observe this effect. In addition, poor matrix–particle interfacial adhesion, which did not enable efficient stress transfer, may have dominated or both micro-scale WP particles may have been generally too large, since this effect appears strongly for nanoscale particle sizes [52]. The reduction in the tensile strength of WP biocomposites can be limited by improving interfacial adhesion through the use of compatibilizers, such as maleic anhydride, as reported by Gowman et al. [32].

Finally, elongation at break is discussed. In general, neat PBS is known for its high flexibility, with an $\varepsilon_{b}$ of around 120%. The results for neat PBS showed high variations with standard deviations of 13% and 17%, comparable to results in the literature [32,33]. As expected, and comparably to conventional composites, as well as other wine by-products, WP filler incorporation reduced $\varepsilon_{b}$ with increasing particle concentration [21,33,34,52]. Due to the higher rigidity of the filler particles and an increasing number of matrix–particle interfaces with poor bonding, crack propagation was promoted, leading to premature failure [52]. This effect can be reinforced by reduced macromolecular chain mobility [32,88]. Compared to RWP-Dom, higher $\varepsilon_{b}$ values were observed for WWP-Silv-based materials. Among all the biocomposites, the sample filled with 10 wt.-% WWP-Silv showed the maximum ductility, reaching the initial $\varepsilon_{b}$ of neat PBS. In most biocomposites, the potential plasticization effects of lipids, such as grape seed oils, are, as described in the literature, mostly overlaid or eliminated by the general reduction in ductility caused by the remaining filler constituents [21]. However, for this specific concentration, the plasticizing effect was remarkable and might have been supported by agglomeration and dispersion phenomena. Apart from that, the mechanics were altered in expected ranges known for conventional polymer composites [52]. From an overall point of view, it is noteworthy that all biocomposites showed smaller standard deviations for each mechanical property compared to neat PBS. This improved predictability of the mechanical properties further confirms the applicability of WP as a functional filler.

## 4. Conclusions

With an increasing demand for sustainable materials and access to existing material streams, especially substantially unused biomass, it is favorable to incorporate biogenic by-products as fillers in biopolyesters, such as biobased PBS. In this way, green biocomposites from renewable resources can contribute to reducing emissions and supporting the shift to sustainable materials. In this study, WP, as the main by-product from the winemaking industry, was investigated not only for use as a low-cost filler in PBS but also as a functional filler to act as a stabilizer while combining economic, sustainable, and technical benefits. Analysis of the composition confirmed the international transferability of the approach, as the constitution of two different varieties of German WP was comparable to WP from other regions in Europe and North America. Furthermore, industrial mill-drying was identified as a viable method for bio-filler preparation. The resulting WP powders were small in particle size with a narrow distribution and showed high total phenolic content. Chemical analysis using spectrophotometric assays revealed high antioxidant and radical scavenging activities for the WP, strengthening the potential for its application as stabilizing functional filler in polymers. Both WP bio-fillers were suitable for PBS-based biocomposites since their thermal stability, investigated using TGA, was sufficient for compounding. Different filler contents of up to 20 wt.-% for both WPs were realized by co-rotating twin-screw extrusion. The resulting biocomposites showed almost unchanged thermal properties and the degree of crystallinity remained constant. TGA measurements showed that WP does not act as a pure thermal stabilizer to suppress β-hydrogen-transfer bond scission. Regarding the main degradation mechanism of PBS, the incorporation of WP led to an outstanding improvement in thermo-oxidative stability. Dynamic OIT analysis revealed that RWP-Dom outperformed WWP-Silv, with optimum stabilization at 5 wt.-% filler content compared to 10 wt.-%. This was consistent with the findings from the chemical analysis using assays. The effect of WP in acting as a thermo-oxidative stabilizer in PBS was verified using TGA measurements in an oxidative atmosphere. Mechanical properties of the biocomposites were altered within expected ranges, as demonstrated by tensile testing. The incorporation of the bio-filler led to an increase in the tensile modulus while reducing the tensile strength and decreasing elongation at break, as is well-known for composite materials. Overall, this study proves that WP, a biogenic by-product generated globally in high amounts, can be valorized for use as a low-cost functional filler in PBS. The stability against thermo-oxidative degradation was increased while maintaining the characteristic thermal properties and altering the mechanical properties within expected ranges. According to the findings from this study, WP filler contents of up to 10 wt.-% are favorable for obtaining biocomposites with balanced thermal, thermo-oxidative, and mechanical properties and additional bio-economic benefits through the valorization of existing biogenic by-products from winemaking. Future research in this area should investigate WP from different years to qualify the potential for sustained use of the materials in industries such as food packaging.

## Figures and Tables

**Table 1:** Temperature profile applied for compounding and brief characterization of the screw configuration in terms of its primary function in relation to its location in the heating zones.

Zone	Die	9	8	7	6	5	4	3	2	1	Feeder
T (°C)	165	165	165	170	170	170	170	170	170	170	170
Screw	Fine dispersing		Transport	degassing	Main mixing		Feeding	Degassing	Kneading		Transport

**Table 2:** Composition of the investigated WPs regarding the shares of their constituents on fresh- and dry-weight bases and the overall RM of the total fresh WP.

By-Product	Skins		Seeds		Stems		RM
	fwb	dwb	fwb	dwb	fwb	dwb	
	(%)	(%)	(%)	(%)	(%)	(%)	(%)
WWP-Silv	58.65 ± 1.00	43.33 ± 1.18	38.64 ± 0.32	54.43 ± 0.90	2.71 ± 0.69	2.24 ± 0.28	61.62 ± 1.22
RWP-Dom	54.79 ± 0.60	44.42 ± 0.77	39.93 ± 1.40	50.70 ± 1.23	5.28 ± 0.80	4.88 ± 0.47	58.25 ± 0.76

fwb: fresh-weight basis; dwb: dry-weight basis; RM: relative moisture.

**Table 3:** Physical properties of the prepared WP fillers regarding particle sizes and relative moisture.

By-Product	$D_{97}$	$D_{50}$	$\bar{D}$	RM
	(µm)	(µm)	(µm)	(%)
WWP-Silv	62.37 ± 0.05	19.45 ± 0.09	23.10 ± 0.07	3.42 ± 0.13
RWP-Dom	51.97 ± 0.09	12.75 ± 0.12	16.80 ± 0.07	2.76 ± 0.18

$D_{97}$: top cut; $D_{50}$: median particle size; $\bar{D}$: mean particle diameter; RM: relative moisture.

**Table 4:** TGA data for prepared WP fillers and determination of characteristic compounds.

By-Product	$T_{5\%}$	$T_{p}$	$∆m_{pectin}$	$∆m_{cellulose}$
	(°C)	(°C)	(%)	(%)
WWP-Silv	198.9	261.7	6.60	22.65
RWP-Dom	230.0	263.4	7.89	26.28

$T_{5\%}$: temperature at 5% mass loss; $T_{p}$: peak temperature of the DTG curve; $∆m_{pectin}$: mass loss in the temperature range ascribed to pectin; $∆m_{cellulose}$: mass loss ascribed to cellulose.

**Table 5:** Chemical properties of native by-products and prepared WP fillers.

By-Product	TPC	DPPH	ABTS
	(mg GAE/g dwb)	(mmol TE/100 g dwb)	(mmol TE/100 g dwb)
WWP-Silv	71.46 ± 1.52	44.45 ± 1.71	55.58 ± 2.46
RWP-Dom	74.73 ± 1.61	51.32 ± 1.35	60.32 ± 2.15

TPC: total phenolic content; DPPH: 2,2-diphenyl-1-picrylhydrazyl assay; ABTS: 2,2′-azinobis-(3-ethylbenzothiazoline-6-sulfonic acid) assay.

**Table 6:** DSC data for neat PBS and WP-based biocomposites.

Filler Content	$T_{m}$	$∆H_{m}$	$T_{c}$	$∆H_{c}$	${Χ}_{c}$
(wt.-%)	(°C)	(%)	(°C)	(%)	(%)
WWP-Silv					
0	117.2 ± 1.2	70.39 ± 0.61	66.1 ± 0.5	69.42 ± 0.82	34.7 ± 0.4
5	117.6 ± 0.7	67.80 ± 0.28	67.3 ± 0.0	64.74 ± 1.18	34.1 ± 0.6
10	116.9 ± 0.6	65.63 ± 1.54	67.8 ± 0.1	63.04 ± 1.46	35.0 ± 0.8
15	117.6 ± 1.1	62.66 ± 0.47	67.3 ± 0.3	61.65 ± 1.83	36.3 ± 1.1
20	116.8 ± 1.5	61.86 ± 1.44	67.1 ± 0.5	58.08 ± 0.59	36.3 ± 0.4
RWP-Dom					
0	117.4 ± 0.8	71.36 ± 2.02	68.8 ± 0.3	68.25 ± 1.53	34.1 ± 0.8
5	116.8 ± 0.3	67.42 ± 0.84	68.8 ± 0.4	65.47 ± 1.17	34.5 ± 0.6
10	116.7 ± 0.6	63.10 ± 1.53	69.3 ± 0.2	62.09 ± 1.49	34.5 ± 0.8
15	117.2 ± 0.8	60.45 ± 1.66	69.6 ± 0.0	57.99 ± 1.52	34.1 ± 0.9
20	116.3 ± 1.3	58.15 ± 1.24	70.1 ± 0.1	55.49 ± 1.82	34.7 ± 1.1

$T_{m}$: melting peak temperature; $∆H_{m}$: melting enthalpy; $T_{c}$: crystallization peak temperature; $∆H_{c}$: crystallization enthalpy; ${Χ}_{c}$: degree of crystallinity.

**Table 7:** TGA data for neat PBS and WP-based biocomposites.

Filler Content (wt.-%)	WWP-Silv			RWP-Dom		
	$T_{10\%}$	$T_{on}$	$T_{p}$	$T_{10\%}$	$T_{on}$	$T_{p}$
	(°C)	(°C)	(°C)	(°C)	(°C)	(°C)
0	362.2 ± 1.2	372.5 ± 1.4	400.2 ± 0.2	362.5 ± 1.1	369.7 ± 0.1	400.4 ± 1.7
5	361.8 ± 0.3	368.9 ± 0.5	394.3 ± 0.7	358.8 ± 0.2	367.1 ± 0.6	392.7 ± 0.5
10	356.6 ± 0.2	364.6 ± 0.6	392.4 ± 0.0	353.8 ± 0.4	364.0 ± 0.8	390.3 ± 0.6
15	353.3 ± 0.9	364.2 ± 0.2	391.5 ± 1.1	347.2 ± 0.6	361.4 ± 0.5	387.4 ± 0.5
20	350.3 ± 0.9	362.7 ± 0.5	390.3 ± 0.1	341.8 ± 0.6	359.3 ± 0.8	388.7 ± 0.3

$T_{10\%}$: temperature at 10% mass loss; $T_{on}$: onset temperature; $T_{p}$: peak temperature (DTG curve).

**Table 8:** Dynamic OIT data for neat PBS and WP-based biocomposites.

Filler Content (wt.-%)	WWP-Silv			RWP-Dom		
	$OIT_{Off}$	$OIT_{On1}$	$OIT_{On2}$	$OIT_{Off}$	$OIT_{On1}$	$OIT_{On2}$
	(°C)	(°C)	(°C)	(°C)	(°C)	(°C)
0	216.7 ± 1.4	243.9 ± 1.6	276.7 ± 0.2	216.5 ± 1.5	248.3 ± 1.2	280.5 ± 2.2
5	262.7 ± 2.4	267.7 ± 0.7	293.4 ± 1.5	277.2 ± 3.5	273.9 ± 0.9	302.1 ± 1.3
10	264.4 ± 7.4	270.5 ± 1.1	294.0 ± 2.5	258.5 ± 6.8	276.2 ± 0.3	295.6 ± 4.2
15	248.8 ± 6.8	269.6 ± 1.1	295.8 ± 1.0	247.3 ± 8.6	275.9 ± 0.2	297.1 ± 1.1
20	243.5 ± 4.1	265.9 ± 0.7	295.3 ± 3.1	237.3 ± 2.5	274.8 ± 1.3	287.7 ± 1.0

$OIT_{\mathrm{Off}}$: dynamic OIT determined with the offset method; $OIT_{On1}$: first-stage dynamic OIT determined with the tangential onset method; $OIT_{On2}$: second-stage dynamic OIT determined with the tangential onset method.

**Table 9:** Oxidative TGA data for neat PBS and WP-based biocomposites.

Filler Content (wt.-%)	WWP-Silv			RWP-Dom		
	$T_{10\%}$	$T_{on}$	$T_{p}$	$T_{10\%}$	$T_{on}$	$T_{p}$
	(°C)	(°C)	(°C)	(°C)	(°C)	(°C)
0	295.6 ± 1.7	293.6 ± 0.9	380.1 ± 0.8	298.3 ± 1.4	295.6 ± 0.4	379.1 ± 0.3
5	336.5 ± 2.1	354.1 ± 1.7	388.4 ± 0.2	341.6 ± 2.4	353.8 ± 0.6	389.0 ± 1.3
10	343.4 ± 0.4	354.9 ± 1.3	389.4 ± 1.7	346.2 ± 1.0	354.3 ± 0.3	387.5 ± 1.7
15	345.7 ± 0.3	357.1 ± 2.8	389.8 ± 0.6	345.1 ± 0.6	357.1 ± 2.0	390.4 ± 0.8
20	333.9 ± 3.5	355.4 ± 1.0	382.7 ± 1.0	339.1 ± 0.7	355.4 ± 1.5	389.3 ± 1.3

$T_{10\%}$: temperature at 10% mass loss; $T_{on}$: onset temperature; $T_{p}$: peak temperature (DTG curve).

**Table 10:** Tensile test data for neat PBS and WP-based biocomposites.

Filler Content (wt.-%)	WWP-Silv			RWP-Dom		
	$E_{t}$	$\sigma_{M}$	$\varepsilon_{b}$	$E_{t}$	$\sigma_{M}$	$\varepsilon_{b}$
	(MPa)	(MPa)	(%)	(MPa)	(MPa)	(%)
0	1073 ± 18	43.3 ± 2.8	113 ± 17	1092 ± 13	46.8 ± 2.1	123 ± 13
5	1124 ± 11	38.8 ± 0.9	97 ± 7	1152 ± 17	38.8 ± 0.6	95 ± 4
10	1166 ± 13	39.7 ± 0.5	115 ± 6	1223 ± 14	34.8 ± 0.4	79 ± 1
15	1206 ± 17	33.7 ± 0.6	85 ± 3	1316 ± 15	31.4 ± 0.2	60 ± 7
20	1257 ± 12	28.2 ± 0.4	45 ± 2	1403 ± 12	29.1 ± 0.2	38 ± 2

$E_{t}$: tensile modulus; *σ_M_*: tensile strength; $\varepsilon_{b}$: elongation at break.

## Data Availability

The data presented in this study are available on request.
